# Effect of miRNA-200a on radiosensitivity of osteosarcoma cells by targeting Bone morphogenetic protein receptor 2

**DOI:** 10.1080/21655979.2021.2011015

**Published:** 2021-12-13

**Authors:** Xian Tao, Jiansheng Cheng, Xinghua Wang

**Affiliations:** Department of Orthopedics, Suzhou Hospital of Integrated Traditional Chinese and Western Medicine, Suzhou, Jiangsu, China

**Keywords:** MiR-200a, BMPR2, osteosarcoma, radiosensitivity, proliferation, apoptosis

## Abstract

To study the effect of miR-200a on radiosensitivity of osteosarcoma cells and its mechanism. NC (normal cell) group, mimic-NC group, mimic-miR-200a group, inhibitor-NC group, inhibitor-miR-200a group, si-NC group, si-BMPR2 (Bone morphogenetic protein receptor 2) group, mimic-miR-200a+vector-NC group, and mimic-miR-200a+vector-BMPR2 group were set; the cells of the above groups were irradiated with different radiation intensities (0, 1, 2, 3, and 4 Gy). The expression of miR-200a and BMPR2 mRNA was detected by qRT-PCR; the expression of BMPR2 protein was detected by Western blot; cell viability was detected by MMT (3-(4,5)-dimethylthiahiazo (-z-y1)-3,5-di-phenytetrazoliumromide); apoptosis rate was detected by flow cytometry. Cell clone formation experiment was used to detect cell radiosensitivity. Dual-luciferase reporter gene test was used to detect cell fluorescence activity. The expression of BMPR2 was high and the expression of miR-200a was low in osteosarcoma tissues after radiotherapy and in osteosarcoma cells after irradiation. Overexpression of miR-200a and interference with BMPR2 expression inhibits osteosarcoma cell proliferation, promotes apoptosis, and increases cellular radiosensitivity, miR-200a targets expression of BMPR2, and overexpression of BMPR2 reverses the radiosensitizing and apoptotic effects of miR-200a expression on osteosarcoma cells. Overexpression of miR-200a inhibits osteosarcoma cell proliferation, promotes apoptosis, and increases cellular radiosensitivity. The mechanism may be related to the regulation of BMPR2, which may provide new targets and new ideas for osteosarcoma treatment.

## 1. Introduction

Osteosarcoma (OS) is one of the most common primary malignant bone tumors in children and adolescents. It has a high degree of malignancy, strong invasiveness, and early metastasis Radiotherapy is one of the main therapeutic methods for osteosarcoma. However, it is of great significance to search for tumor radiosensitizers because of its poor efficacy due to the emergence of drug resistance and radiosensitivity [1,2]. MicroRNA (miRNA) is a class of non-coding small molecule RNA that is widely involved in cell proliferation, differentiation, apoptosis, and regulation of tumor radiosensitivity, and miRNAs are also associated with the development, progression, and metastasis of osteosarcoma [3,4]. miR-200a was found to be poorly expressed in osteosarcoma tissues and osteosarcoma cell lines, and may play an important role in the development, metastasis, and survival of osteosarcoma [5]. Abnormal differential expression of miRNA-200a in cervical squamous cell carcinoma before and after radiotherapy may serve as a biomarker for cervical squamous cell carcinoma after radiotherapy [6]. Overexpression of miR-200a increases radiosensitivity in non-small cell lung cancer [7]. Bone morphogenetic protein (BMP) is highly expressed in osteosarcoma cells and promotes the proliferation of tumor cells. BMPR2 (Bone morphogenetic protein receptor 2) is a receptor of BMP and is also highly expressed in osteosarcoma cells [8]. Inhibition of BMPR2 expression enhances the sensitivity of glioma stem cells to differentiated therapies [9]. However, it is unclear whether the effect of miR-200a on the radiosensitivity of osteosarcoma cells and its mechanism is related to BMPR2, and the aim of this study is to investigate whether miR-200a affects the radiosensitivity of osteosarcoma cells by modulating BMPR2. To provide new targets and new ideas for radiotherapy of osteosarcoma cells.

## 2. Material and methods

### 2.1 Materials and Reagents

Osteosarcoma cell MG-63 and U2-OS were purchased from Shanghai Cell Bank of Chinese Academy of Sciences, and osteosarcoma tissue before and after radiotherapy was obtained from local Cancer Hospital. Fetal bovine serum and DMEM (dulbecco’s modified eagle medium) culture were purchased from Gibco, USA; RNA extraction kit, reverse transcription kit and qRT-PCR (Quantitative Real-time Polymerase Chain Reaction) kit were purchased from Takara, Japan; Lipofectamine^TM^ 2000 transfection kit was purchased from Invitrogen, USA; MTT (3-(4,5)-dimethylthiahiazo (-z-y1)-3,5-di- phenytetrazoliumromide) kit, Annexin V-FITC kit and propidium iodide (PI) kit, dual luciferase reporter gene assay kit were purchased from Beyotime Biotech Inc., Shanghai; Dimethyl sulfoxide (DMSO), BCA kit, RIPA protein lysate, and SDS-PAGE kit were purchased from Sigma; Co60 medical irradiation device was purchased from Nuclear Power Institute of China.

### 2.2 Methods

#### 2.2.1 Cell culture

Osteosarcoma cell line MG-63 and U2-OS were cultured in DMEM supplemented with 10% FBS (Fetal bovine serum) at 37°C in an incubator containing 5% CO_2_ and subcultured every 2 ~ 3 days. The logarithmically growing cells were used in the experiment.

#### 2.2.2 Transfection and grouping of cells

Osteosarcoma cells MG-63 and U2-OS were inoculated into 6-well plate, respectively after digestion. When the cells grew to 80% fusion, they were replaced with serum-free medium for 12 hours, and then transfected. The overexpression plasmid, inhibition plasmid and control plasmid of miR-200a, interfering plasmid and negative control plasmid of BMPR2 were transfected into MG-63 cells, which were classified as mimic-NC group, mimic-miR-200a group, inhibitor-NC group, inhibitor-miR-200a group, si-NC group, and si-BMPR2 group, respectively. miR-200a overexpression plasmid, BMPR2 overexpression plasmid, and BMPR2 overexpression control plasmid were co-transfected into MG-63 cells, which were classified as mimic-miR-200a+vector-NC group and mimic-miR-200a+vector-BMPR2 group, respectively. MG-63 cells without any treatment were used as blank control (NC) group. The cell transfection was performed using Lipofectamine^TM^ 2000 kit according to the manufacturer’s instructions. The NC, mimic-NC, mimic-miR-200a, si-NC, si-BMPR2, mimic-miR-200a+vector-NC, and mimic-miR-200a+vector-BMPR2 group cells were irradiated with 0 Gy, 1 Gy, 2 Gy, 3 Gy, and 4 Gy, respectively as different dose irradiation group, and unexposed cells served as normal controls.

#### 2.2.3 miR-200a and BMPR2 mRNA expression analyzed by qRT-PCR

Total RNA of cells was extracted according to Trizol instructions, reversely transcribed into cDNA using reverse transcription kit, and amplified according to fluorescence quantitative instructions. Cycle conditions are 95°C 30s, 60°C 30s, 72°C 30s, 40 cycles in total; 60°C extended for 5 min. Relative expression is calculated by using 2^−ΔΔCt^ method [10].

#### 2.2.4 BMPR2 protein expression by Western blot

The cells of Inhibitor-NC group, inhibitor-miR-200a group, mimic-NC group, and mimic-miR-200a group were collected, and lysed with RIPA lytic solution, centrifuged at 12,000 g for 15 min at 4°C. The supernatant was collected for protein concentration quantification with BCA Protein Assay Kit After SDS-PAGE (sodium dodecyl sulfate polyacrylamide gel electropheresis) electrophoresis, the protein sample was transferred to PVDF (polyvinylidencefluoride) membrane. Then the PVDF membrane was blocked with 5% skimmed milk powder blocking solution at room temperature (22–24°C) for 1 h. The primary antibody (Anti-BMPR1B antibody, 1:1000, ab175385; Anti-GAPDH antibody, 1:2500, ab9485) were added, incubated at 4°C overnight. TBST was used to wash the membrane. the secondary antibody (Fluorescein-labeled Goat Anti-Rabbit (1:2000, ab6721) was added at room temperature (22–24°C) for 2 h, and the TBST was washed for 3 times, each time for 10 min. The above antibodies were purchased from Abcam (MA, USA). The protein samples were exposed and developed in the darkroom, then immersed in the fixing solution for fixing, and finally washed away the residual solution and dried. The film was processed with Quantity One gel analysis software, the absorbance of each protein band was determined. The ratio of the target band and the GAPDH (glyceraldehyde-3-phosphate dehydrogenase) band was used as the protein expression level [11]. Each protein sample was repeated 3 times.

#### 2.2.5 Cell viability by MTT assay

After irradiation, 20 μL (5 g/L) MTT solution was added to each group of cells and normal cell culture up to 48 h, and the incubation continued for 4 h. Redundant medium was discarded, and 150 μL DMSO (Dimethyl sulfoxide) was added for shaking reaction for 10 min, and absorbance (OD) value at 490 nm was detected by microplate reader. Cell viability (%) = OD value of experimental group/OD value of blank control group × 100% [12].

#### 2.2.6 Apoptosis rate by flow cytometry

After irradiation, each group of cells and normal cells were digested with pancreatic enzyme without EDTA (Ethylene Diamine Tetraacetic Acid), collected by centrifugation, rinsed twice with PBS, and resuspended with binding buffer. Incubate with Annexin V-FITC and PI protected from light according to the kit instructions. Flow cytometry detects fluorescence intensity at an excitation wavelength of 488 nm and an emission wavelength of 530 nm [13]. The experiment was repeated 3 times.

#### 2.2.7 Cell colony formation assay

The cells of mimic-NC, mimic-miR-200a, si-NC, si-BMPR2, mimic-miR-200a + vector-NC, mimic-miR-200a + vector-BMPR2 groups were inoculated into culture dishes with a diameter of 60 mm at proper density. The cells were fused to about 80% and irradiated with 0 Gy, 1 Gy, 2 Gy, 3 Gy, and 4 Gy. After irradiation, 0.3 × 10^3^, 1 × 10^3^, 2 × 10^3^, 3 × 10^3^, and 4 × 10^3^ cells were inoculated into 60 mm culture dishes according to the dose. The culture was continued for 10–14 days. The culture dishes were taken out and cleaned twice with PBS, fixed with methanol for 15 min, and stained with Giemsa for 30 min. Colonies of > 50 cells were counted under low-power light microscopy. Planting efficiency (PE) = number of clones/number of cells inoculated × 100%, survival fraction (SF2) = number of colonies in irradiation dose group/(number of cells inoculated in this group × PE in non-irradiation group). According to the multi-target single-hit model [SF = 1- (1-e-D/D_0_) N, D_q_ = D_0_ × lnN.], a cell survival curve was drawn for calculating the sensitization enhancement ratio (SER). Where D is the irradiation dose (Gy), D_0_ is the average lethal dose, D_q_ is the threshold dose (represents the wide shoulder of survival), and N is the extrapolated value. SER = D_0_ in simple sensitization group/D_0_ in combined irradiation group [14].

#### 2.2.8 Detection of BMPR2 targeting by miR-200a using luciferase reporter assay

The TargetScan database shows the miR-200a binding site in the BMPR2 3ʹ-UTR region. The 3ʹ-UTR luciferase expression vectors (BMPR2-wt and BMPR2-mut) of wild-type and mutant gene target BMPR2 were constructed. MG-63 cells in logarithmic growth phase were inoculated on 24-well plate (1 × 10^3^/well). When the cells grew to 80% fusion, mimic-NC and mimic-miR-200a plasmids were transfected into BMPR2-wt and BMPR2-mut cells with Lipofectamine^TM^ 2000 respectively. According to the instructions, double luciferase reporter assay was carried out by using luciferase reporter gene detector. The results were analyzed statistically as the ratio of luciferase activity to Renilla activity [15]. The experiment was repeated 3 times.

#### 2.2.9 Statistical analysis

GraghPad Prism5 was used to fit the cell survival curves. SPSS 20.00 was used for statistical analysis. Measurement data were expressed as x ± s. t-test was performed for comparison between two groups [16]. Single factor analysis of variance was used for comparison among multiple groups [17]. P < 0.05 was used for statistical significance.

## 3. Results

In order to explore whether miR-200a affects the radiosensitivity of osteosarcoma cells by regulating BMPR2, and to provide new targets and new ideas for radiotherapy of osteosarcoma cells. We observed the expression levels of BMPR2 and miR-200a in osteosarcoma tissues and cell lines before and after radiotherapy, and further studied the effects of miR-200a and BMPR2 on the sensitivity of osteosarcoma cells to radiotherapy.

### 3.1 Expression levels of BMPR2 and miR-200a in osteosarcoma tissues before and after radiotherapy

The results of qRT-PCR (Table 1) showed that the expression level of BMPR2 was significantly higher and the expression level of miR-200a was significantly lower in osteosarcoma tissues after radiotherapy than that before radiotherapy (P < 0.05).

**Table 1.** Expression of BMPR2 and miR-200a before and after radiotherapy

Osteosarcoma tissue	Sample	miR-200a	BMPR2
Before radiotherapy	13	0.98 ± 0.094	1.31 ± 0.124
After radiotherapy	13	0.51 ± 0.049^a^	1.71 ± 0.164^a^
*t*		15.986	7.015
*P*		0.000	0.000

Notes: aP<0.05 vs before radiotherapy

### 3.2 Expression of miR-200a and BMPR2 in osteosarcoma cell lines

The results of qRT-PCR (Tables 2 and 3) showed that the expression level of BMPR2 was significantly higher and the expression level of miR-200a was significantly lower in the osteosarcoma cell line (MG-63 and U2-OS) after 2 Gy radiotherapy than in the osteosarcoma cell line (MG-63 and U2-OS) before radiotherapy (P < 0.05). In the following experiment, we used MG-63 for research.

**Table 2.** Expression of miR-200a and BMPR2 in MG-63 cell lines

Osteosarcoma cell line	miR-200a	BMPR2
0 Gy+MG-63	0.96 ± 0.097	1.17 ± 0.114
2 Gy+MG-63	0.47 ± 0.050	1.69 ± 0.160
*t*	13.470	7.941
*P*	0.000	0.000

**Table 3.** Expression of miR-200a and BMPR2 in U2-OS cell lines

Osteosarcoma cell line	miR-200a	BMPR2
0 Gy+U2-OS	1.0 ± 0.10	1.10 ± 0.10
2 Gy+U2-OS	0.53 ± 0.05	1.70 ± 0.10
*t*	12.610	12.730
*P*	0.000	0.000

### 3.3 Effect of miR-200a on radiosensitivity of osteosarcoma cells in vitro

**Figure 1.**
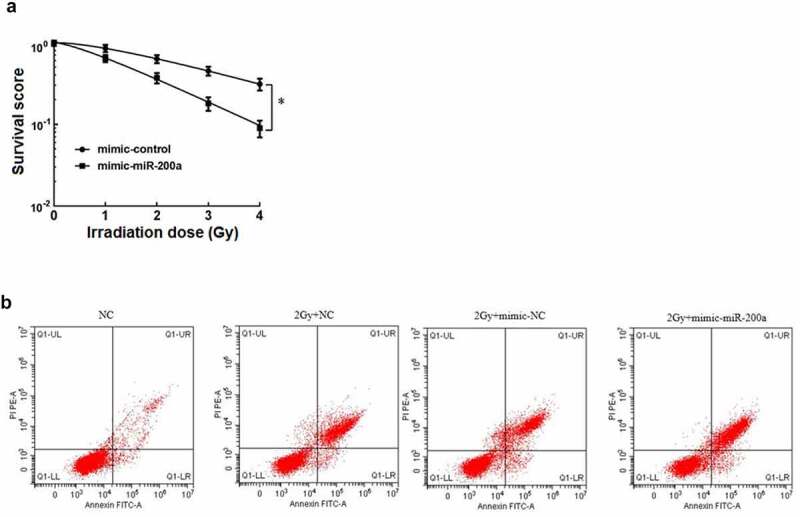
Effect of miR-200a on radiosensitivity of osteosarcoma cells in vitro. a: Single-hit multi-target model to fit the cell survival curve. b: Overexpression of mimic-200a by 2 Gy radiotherapy for apoptosis of osteosarcoma cells

After cell clone formation experiment, the sensitization enhancement ratio (SER) of mimic-miR-200a group was 1.637 by single-hit multi-target model fitting (Table 4). The cell survival curve (Figure 1(a)) showed that the cell survival curve of mimic-miR-200a group moved down significantly with the increase of irradiation dose. Osteosarcoma cell viability was significantly reduced with increasing doses of mimic-miR-200a compared with those in the mimic-NC group (P < 0.05, Table 5). Osteosarcoma cell death rate was significantly higher in the 2 Gy+NC group than in the NC group, and osteosarcoma cell death rate was also significantly higher in the 2 Gy+mimic-NC group than that in 2 Gy+mimic-miR-200a group (P < 0.05, Table 6). It can be seen that overexpression of miR-200a increases the radiosensitivity of osteosarcoma cells in vitro, inhibits cell survival, and promotes apoptosis.

**Table 4.** Effect of overexpression of mimic-200a on Single-hit multi-target model parameters and mimic-200a in MG-63 cells

Group	Sample	Single-hit multi-target model parameter						Expression of miR-200a
		D_0_(Gy)	D_q_(Gy)	N	SF2	k	SER	
mimic-NC	9	2.425	1.339	1.737	0.633	0.412	-	0.71 ± 0.065
mimic-miR-200a	9	1.481	0.563	1.462	0.355	0.675	1.637	1.27 ± 0.121^a^
*t*								12.231
*P*								0.000

Notes: ‘-’- is no data; ^a^*P*<0.05 *vs* mimic-NC

**Table 5.** Effects of overexpression of miR-200a on the viability of osteosarcoma cells during different doses of radiotherapy

Group	0 Gy	1 Gy	2 Gy	3 Gy	4 Gy
NC	1.09 ± 0.091	0.85 ± 0.068	0.74 ± 0.056	0.50 ± 0.045	0.41 ± 0.046
mimic-NC	1.06 ± 0.096	0.87 ± 0.085	0.72 ± 0.076	0.48 ± 0.040	0.39 ± 0.041
mimic-miR-200a	1.07 ± 0.110	0.76 ± 0.078^a^	0.65 ± 0.061^a^	0.38 ± 0.036^a^	0.19 ± 0.021^a^
*F*	0.213	5.169	4.773	22.678	94.290
*P*	0.810	0.014	0.018	0.000	0.000

Notes: aP<0.05 vs mimic-NC

**Table 6.** Effect of overexpression of miR-200a on mortality of osteosarcoma cells

Group	Death rate of osteosarcoma cells
NC	7.45 ± 0.831
2 Gy+NC	31.62 ± 3.450^a^
2 Gy+mimic-NC	32.77 ± 3.165
2 Gy+mimic-miR-200a	46.71 ± 4.127^b^
*F*	241.408
*P*	0.000

Notes: ^a^*P*<0.05 vs NC; ^b^*P*<0.05 *vs* 2 Gy+mimic-NC

### 3.4 miR-200a can bind to BMPR2

**Figure 2.**
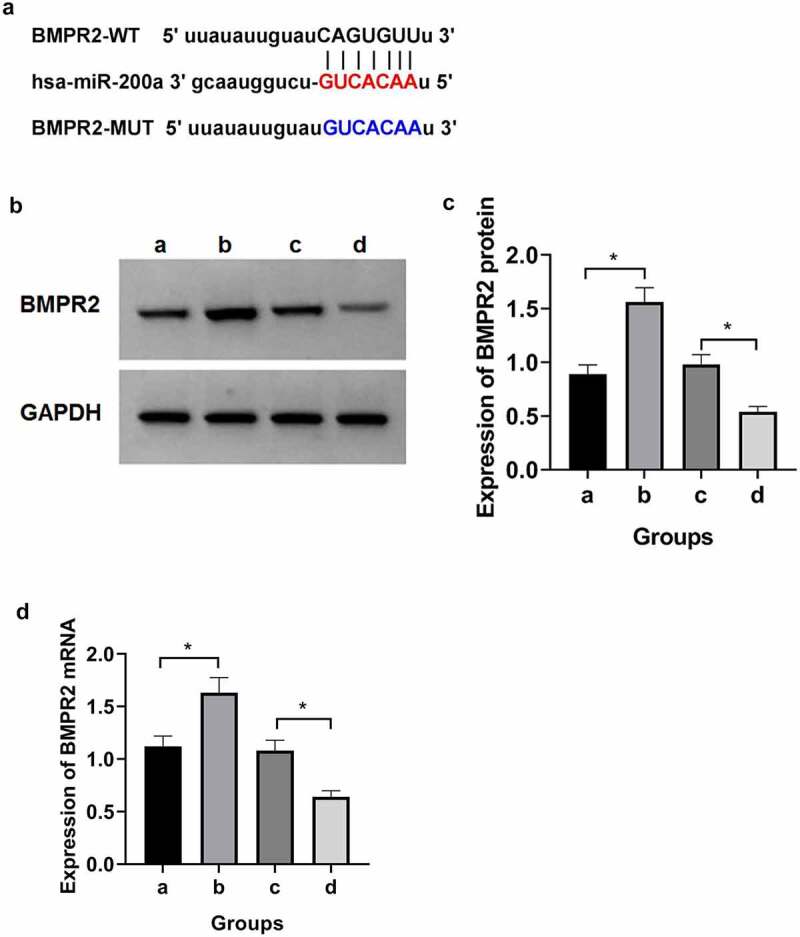
miR-200a targeted regulation of BMPR2. a: Binding site of BMPR2 and miR-200a. b: Western blot. c:Expression of BMPR2 protein when overexpressing or inhibiting miR-200a. d:Expression of BMPR2 miRNA when overexpressing or inhibiting miR-200a. a:inhibitor-NC; b:inhibitor-miR-200a; c:mimic-NC; d:mimic-miR-200a

The BMPR2 binding site to miR-200a was predicted from the TargetScan database (Figure 2(a)). The luciferase reporter assay results (Table 7) showed that compared with BMPR2-wt+mimic-NC group, the amount of luciferase expression in osteosarcoma cells was significantly reduced in BMPR2-wt+mimic-miR-200a group (P < 0.05), whereas the amount of luciferase expression in osteosarcoma cells in BMPR2-mut+mimic-miR-200a was not significantly different from that in BMPR2-mut+mimic-NC group. Results of qRT-PCR and Western blot assays (Figure 2(b), Table 8) showed that BMPR2 expression was significantly higher in osteosarcoma cells in inhibitor-miR-200a group than that in inhibitor-NC group; BMPR2 expression was significantly lower in osteosarcoma cells in mimic-miR-200a group than in those in mimic-NC group (P < 0.05). It can be seen that miR-200a targets BMPR2 expression.

**Table 7.** Dual luciferase reporting experiment

Group	Fluorescence expression
BMPR2-wt+mimic-NC	1.08 ± 0.098
BMPR2-wt+mimic-miR-200a	0.57 ± 0.051^a^
BMPR2-mut+mimic-NC	1.10 ± 0.097
BMPR2-mut+mimic-miR-200a	1.12 ± 0.105
*F*	77.751
*P*	0.000

Notes: ^a^*P*<0.05 *vs* BMPR2-wt+mimic-NC

**Table 8.** Effect of miR-200a expression on BMPR2

Group	BMPR2 mRNA expression	Protein expression of BMPR2
inhibitor-NC	1.12 ± 0.098	0.89 ± 0.085
inhibitor-miR-200a	1.63 ± 0.144^a^	1.56 ± 0.134^a^
mimic-NC	1.08 ± 0.097	0.98 ± 0.091
mimic-miR-200a	0.64 ± 0.058^b^	0.54 ± 0.049^b^
*F*	136.963	179.843
*P*	0.000	0.000

Notes: ^a^*P*<0.05 *vs* inhibitor-NC; ^b^*P*<0.05 *vs* mimic-NC;

### 3.5 Effect of BMPR2 on radiosensitivity of osteosarcoma cells

**Figure 3.**
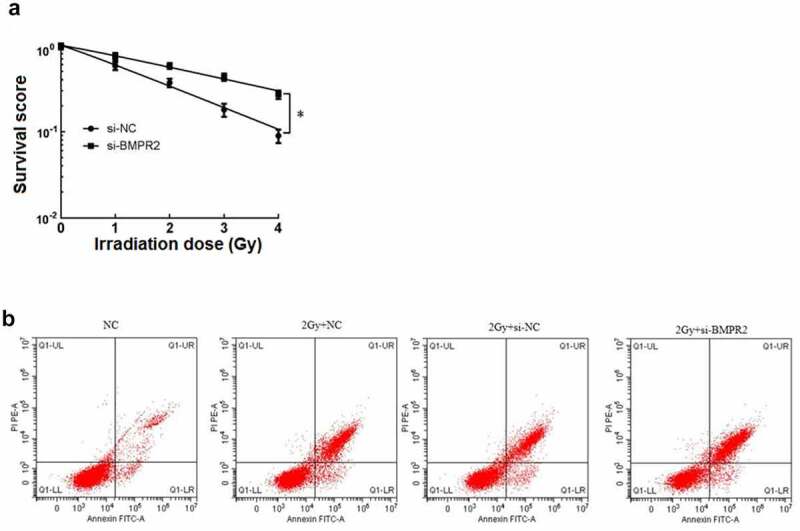
Effect of BMPR2 on radiosensitivity of osteosarcoma cells.a: Single-hit multi-target model to fit the cell survival curve. b: Effect of flow cytometry on osteosarcoma cells after 2 Gy radiotherapy irradiation when interfering with BMPR2

After cell clone formation experiment, the sensitization enhancement ratio (SER) of si-BMPR2 group was 0.559 by single-hit multi-target model fitting (Table 9). The cell survival curve (Figure 3(a)) showed that the survival curve of si-BMPR2 group decreased significantly with the increase of irradiation dose. Compared with NC group, 2 Gy+NC group had significantly lower osteosarcoma cell viability and higher death rate. Compared with 2 Gy+si-NC group, 2 Gy+si-BMPR2 group had significantly lower osteosarcoma cell viability and higher death rate (P < 0.05, Table 10). The results showed that BMPR2 could increase the radiosensitivity of osteosarcoma cells, inhibit cell survival and promote apoptosis.

**Table 9.** Effect of BMPR2 on multi-target model parameters and BMPR2 in MG-63 cells

		Single-hit multi-target model parameter						
Group	Sample	D0(Gy)	Dq(Gy)	N	SF2	k	SER	Expression of BMPR2
si-NC	9	1.717	0.168	1.103	0.338	0.583	-	1.15 ± 0.105
si-BMPR2	9	3.069	0.334	1.115	0.560	0.326	0.559	0.56 ± 0.051^a^
*t*								15.163
*P*								0.000

Notes:‘-’is no data; ^a^*P*<0.05 *vs* si-NC

**Table 10.** Effects of interfering with BMPR2 on cell viability and mortality

Group	Osteosarcoma cell viability	Death rate of osteosarcoma cells
NC	1.12 ± 0.102	9.30 ± 0.891
2 Gy+NC	0.66 ± 0.062^a^	34.21 ± 3.245^a^
2 Gy+si-NC	0.62 ± 0.058	32.17 ± 3.151
2 Gy+si-BMPR2	0.31 ± 0.026^b^	48.64 ± 4.210^b^
*F*	219.472	244.363
*P*	0.000	0.000

Notes: ^a^*P*<0.05 *vs* NC; ^b^*P*<0.05 *vs* 2 Gy+si-NC

### 3.6 miR-200a enhances radiosensitivity of osteosarcoma cells by targeting BMPR2 expression

**Figure 4.**
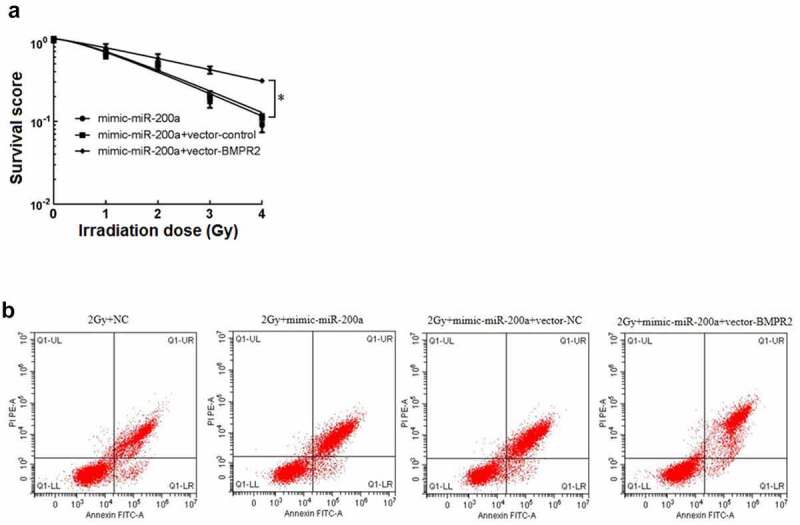
Effect of miR-200a on osteosarcoma cells by targeted regulation of BMPR2. A: Click on the multi-target model to fit the cell survival curve. B: Effect of flow cytometry on osteosarcoma cells after 2 Gy radiotherapy with overexpression of miR-200a and BMPR2

After cell clone formation experiment, the sensitization enhancement ratio (SER) of mimic-miR-200a+vector-BMPR2 group was 0.538 calculated by single-hit multi-target model fitting (Table 11). The cell survival curve (Figure 4a) showed that the cell survival curve of mimic-miR-200a+vector-BMPR2 group moved up significantly with the increase of irradiation dose. There was a significant decrease in osteosarcoma cell viability and a significant death rate in the 2 Gy+mimic-miR-200a group compared with those in the 2 Gy+NC group, and a significant increase in osteosarcoma cell viability and a significant death rate in the 2 Gy+mimic-miR-200a+vector-BMPR2 group compared with those in the 2 Gy+mimic-miR-200a+vector-NC group (P < 0.05, Figure 4b, Table 12). Overexpression of BMPR2 reverses the effects of miR-200a on radiosensitization, proliferation inhibition, and apoptosis in osteosarcoma cells.

**Table 11.** Effect of BMPR2 inhibition on Single-hit multi-target model parameters and BMPR2 in MG-63 cells

Group	Sample	Single-hit multi-target model parameter					
		D_0_(Gy)	D_q_(Gy)	N	SF2	k	SER
mimic-miR-200a	9	1.558	0.682	1.549	0.395	0.642	-
mimic-miR-200a+vector-NC	9	1.617	0.722	1.563	0.415	0.619	-
mimic-miR-200a+vector-BMPR2	9	3.004	0.545	1.199	0.579	0.333	0.538

Notes: ‘-’ is no data;

**Table 12.** mRNA expression of BMPR2 and cell viability and mortality

Group	BMPR2 mRNA	Cell viability	Cell death rate
2 Gy+NC	1.09 ± 0.099	0.72 ± 0.068	32.87 ± 3.014
2 Gy+mimic-miR-200a	0.42 ± 0.039^a^	0.38 ± 0.032^a^	56.35 ± 3.540^a^
2 Gy+mimic-miR-200a+vector-NC	0.48 ± 0.042	0.40 ± 0.037	54.69 ± 5.540
2 Gy+mimic-miR-200a+vector-BMPR2	0.71 ± 0.062^b^	0.52 ± 0.048^b^	42.45 ± 4.088^b^
*F*	195.984	94.110	63.677
*P*	0.000	0.000	0.000

Notes: ^a^*P*<0.05 *vs* 2 Gy+NC; ^b^*P*<0.05 *vs* 2 Gy+mimic-miR-200a+vector-NC

## 4. Discussion

Radiotherapy and chemotherapy are conservative treatment means for primary lesions. Bone metastases from primary bone tumors have a poor prognosis, but radiotherapy can help control the development of the disease. However, osteosarcoma cells are radioresistant and new and more effective radiosensitizers are needed to enhance the effect of radiotherapy [18]. Some miRNAs have been shown to play an important role in the radiosensitivity of osteosarcoma, and miRNAs have the potential to become new targets targeting increased radiosensitivity of osteosarcoma by modulating related genes, increasing the rate of apoptosis, and reducing tumor radiation resistance [19]. miR-200a and miR-200 c are both belong to the microRNA 200 family, miR-200 c has been shown to enhance radiosensitivity in breast cancer cells by targeting UBQLN1 [20]; miR-200 c also enhances radiosensitivity in lung cancer [21]. In contrast, studies of the radiosensitivity of miR-200a to tumor cells have reported that miR-200a is highly expressed in radioresistant oral squamous cell carcinoma cells and is associated with radioresistance in oral squamous cell carcinoma [22]. miR-200a is significantly under-expressed in osteosarcoma tissues and inhibition of miR-200a promotes the invasion and migration of osteosarcoma cells [23]. Here, we show that miR-200a is poorly expressed in radiation-treated osteosarcoma tissue and radiation-irradiated osteosarcoma cells, and overexpression of miR-200a suppresses osteosarcoma cell proliferation, promotes apoptosis, and enhances cellular radiosensitivity.

BBMPR is a transmembrane serine/threonine protein kinase receptor of the TGFβ receptor superfamily. The corresponding ligand is bone morphogenetic proteins (BMP). BMPR2 is involved in bone repair, bone remodeling and cell migration and apoptosis [24]. Studies have shown that BMPR2 is highly expressed in most osteosarcoma tissues, is associated with overall survival in osteosarcoma patients, and promotes invasion and metastasis via the RhoA-ROCK-LIMK2 pathway in human osteosarcoma cells [25]. Therefore, we speculated that the downstream pathway of miR-200a-BMPR2 is RhoA-ROCK-LIMK2 pathway, but further experimental proof is still needed. Inhibition of BMPR2 induces apoptosis and autophagy in human chondrosarcoma by destabilization of XIAP [26]. BMPR2 has a tumor suppressor function in the mammary epithelium and microenvironment, and its disruption accelerates breast cancer metastasis [27]. Our results suggest that BMPR2 is overexpressed in radiation-treated osteosarcoma tissue and radiation-irradiated osteosarcoma cells, interfering with BMPR2 expression inhibits osteosarcoma cell proliferation, promotes apoptosis, and increases cell radiosensitivity. These results suggest that BMPR2 is not only associated with the invasion, metastasis and apoptosis of osteosarcoma cells, but also regulates the radiosensitivity of cells. Through a previous literature search, it was found that the BMPR2 has been shown to be related to the invasion and metastasis of human osteosarcoma cells. In addition, we predicted the binding site of BMPR2 and miR-200a through the TargetScan database, indicating that BMPR2 is the target of miR-200a, so we studied the regulatory effect of miR-200a on BMPR2. We found that miR-200a targets BMPR2 expression, and overexpression of BMPR2 reverses the effects of miR-200a expression on radiosensitization and induction of apoptosis in osteosarcoma cells. This suggests that miR-200a may influence the proliferation, apoptosis, and radiosensitivity of osteosarcoma cells by modulating BMPR2.

## 5 Conclusion

Overexpression of miR-200a inhibits osteosarcoma cell proliferation, promotes apoptosis, and increases cellular radiosensitivity, its mechanism may be involved in the regulation of BMPR2, and may provide new targets and new ideas for radiation therapy of osteosarcoma.
